# Open surgical excision vs. endoscopic radiofrequency ablation in managing congenital pyriform sinus fistula—a comprehensive analysis of 166 cases

**DOI:** 10.3389/fped.2024.1387626

**Published:** 2024-07-22

**Authors:** Mengrou Xu, Wei Chen, Jiarui Chen, Xiaoyan Li

**Affiliations:** Department of Otorhinolaryngology Head and Neck Surgery, Shanghai Children’s Hospital, Shanghai Jiao Tong University School of Medicine, Shanghai, China

**Keywords:** congenital pyriform sinus fistula, children, open surgical excision, endoscopic radiofrequency ablation, neck abscess

## Abstract

**Background:**

Congenital pyriform sinus fistula (CPSF) is uncommon congenital abnormality, and the optimal definitive treatment has not yet been established. The aim of the present study was to comparatively evaluate patients with CPSF regarding the indications and outcomes of treatment with open surgical excision (OSE) vs. endoscopic Radiofrequency Ablation (RA), and the advantages of both therapeutic procedures were also analyzed.

**Methods:**

An observational, longitudinal, retrospective, analytical and comparative study was conducted on 166 consecutive pediatric patients with CPSF treated at Shanghai Children's Hospital between December 2018 and September 2023.

**Results:**

In this study, there were 79 males and 87 females. The median age at operation was 4.8 years (8 days to 15 years). OSE and Endoscopic RA were respectively performed in 48 and 118 children. The gastric tube retention time after RA was longer (3 days vs. 14 days) than after OSE. Patients with a history of incision and drainage (I&D) tended to choose OSE (75.0% vs. 39.0%, *P *< 0.01). There were no significant differences in postoperative complications and recurrence rates between these two groups (*P *> 0.05), but the hospitalization duration was shorter for RA group compared to OSE group [2 (2–3) vs. 4 (3–5), *P *< 0.01].

**Conclusions:**

Both OSE and RA are recognized as first-line treatment options for CPSF, which show no significant differences in prognosis, except hospitalization duration and the gastric tube retention time. Nevertheless, the indications for OSE and RA differ, which are influenced by factors such as the inflammatory stage, specific typing, previous treatments, and the surgeon's expertise. The selection of surgical approach should be carefully determined based on individual circumstances.

## 1 Introduction

Congenital pyriform sinus fistula (CPSF) is a rare branchiogenous disease of the neck, which is known as the third or fourth branchial cleft anomaly. According to the direction of fistula, third and fourth branchial cleft can be distinguished. Third branchial cleft originates from the base of the pyriform fossa, then passes through the thyrohyoid membrane, and is located above the superior laryngeal nerve (1). Fourth branchial cleft originates from the tip of the pyriform fossa and passes through the cricothyroid membrane, located below the superior laryngeal nerve (2). As the clinical manifestations and treatment methods for third and fourth branchial cleft are the same, they are collectively referred to pyriform sinus fistula. The primary clinical manifestations of CPSF include recurrent neck swelling, neck abscess, suppurative thyroiditis or subacute thyroiditis (3). Due to its rarity and close association with thyroid gland, it is often overlooked and misdiagnosed as thyroiditis (4), which brings diagnostic and therapeutic challenges. Approximately 90% of the fistula occurs on the left side, with occasional instances on the right side and bilaterally (5).

For the treatment of CPSF in acute infection stage (AIS), conventional approaches include antibiotic treatment, incision and drainage, or puncture drainage. Over the years, traditional open surgery was commonly used to remove the entire fistulous tract (3, 6), but in recent years, with the advancement of minimally invasive techniques, endoscopic cauterization of the internal opening has become increasingly popular, including Radiofrequency Ablation (RA), laser coagulation, and others (5, 7).

In this study, we collected data from patients with CPSF and evaluated the indications and outcomes of open surgical excision (OSE) vs. RA. The aim of the present study was to comparatively evaluate patients with CPSF regarding the indications and outcomes of treatment with OSE vs. endoscopic RA, and the advantages of both therapeutic procedures were also analyzed.

## 2 Patients and methods

### 2.1 General data

In this observational, longitudinal, retrospective, analytical and comparative study, we reviewed 166 patients with CPSF who received treatment at Shanghai Children's Hospital from December 2018 to September 2023. Prior to data collection, we obtained approval from the institution's research ethics board. The following data were collected from these children: gender, age, side, typical symptoms, infection condition, imaging examinations, surgical methods, previous treatments, length of hospital stay, postoperative complications, recurrence as well as length of follow-up. The respective indications of OSE and endoscopic RA depend mainly on the basic situation of the patient. OSE was adopted in the following situations: (1) Giant pyriform fossa cyst of newborn; (2) Obvious scar in the neck following previous unsuccessful incision and drainage (I&D) or open surgical procedures; (3) Patients with recurrence after RA surgery. In addition, due to the minimally invasive nature of endoscopic RA, some patients still choose RA surgery after recurrence. Contraindication for OSE is: CPSF patients in the AIS. For patients with acute infection and neck abscess formation, based on previous experience, our department recommends RA and I&D of neck abscesses simultaneously. Indications, treatment outcomes, and advantages of OSE vs. endoscopic RA were compared.

### 2.2 Surgical methods

#### 2.2.1 Endoscopic RA

**Figure 1:**
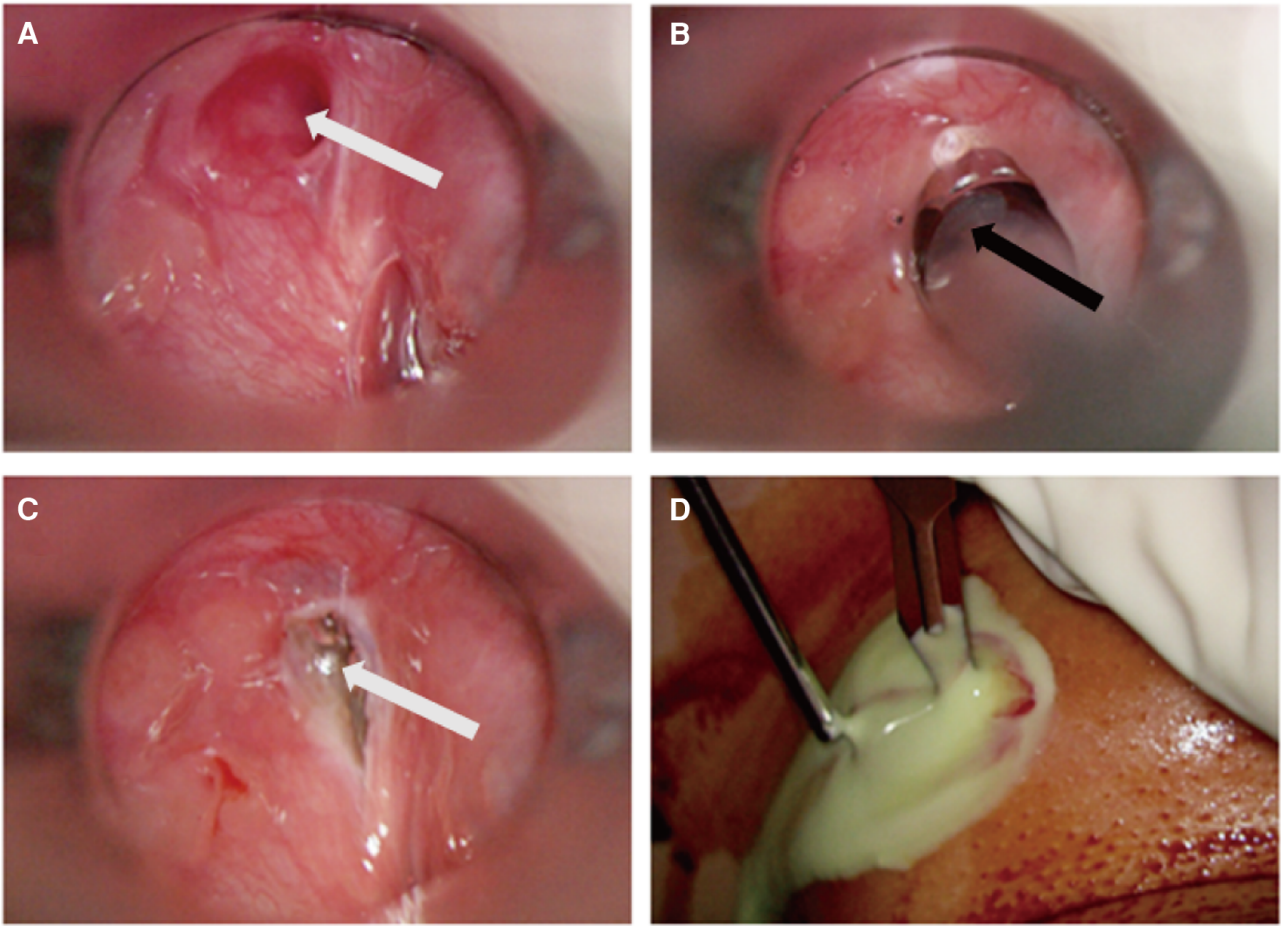
Intraoperative images of endoscopic RA. (**A**) The internal opening of the fistulous tract; (**B**): low-temperature plasma electrode in the sinus tract during R; (**C**): view after RA. White arrow: the internal opening of CPSF; Black arrow: plasma electrode (**D**): I&D of neck abscess in combination with RA in AIS. CPSF: congenital pyriform sinus fistula; RA: radiofrequency ablation; AIS: acute infection stage.

Endoscopic RA for CPSF was performed by the chief surgeon (Dr. Li). The patients were placed in the supine position and underwent tracheal intubation under general anesthesia. The internal opening of the CPSF was identified by a suspension laryngoscope (OVT-S7V; Olympus, Tokyo, Japan). Diluted iodine was used to disinfect the internal opening and surrounding mucosa. And then, RA (EIC7070-01, American ArthroCare Corporation) was used to ablate the sinus tract from distal end to proximal end, including the tract located on the interior part of the thyroid cartilage. The coblation technique was utilized to address the fistula, which measured approximately 0.5 cm in diameter. The procedure involved targeting the area surrounding the fistula with precision, ensuring that the depth of the coblation did not surpass the level of the thyroid cartilage plate (Figure 1). The power was maintained at 7–10 W. For 35 patients in AIS, I&D of neck abscesses were performed simultaneously. After undergoing RA surgery, patients usually have a nasogastric tube retained for a duration of 14 days.

#### 2.2.2 Open surgical excision

**Figure 2:**
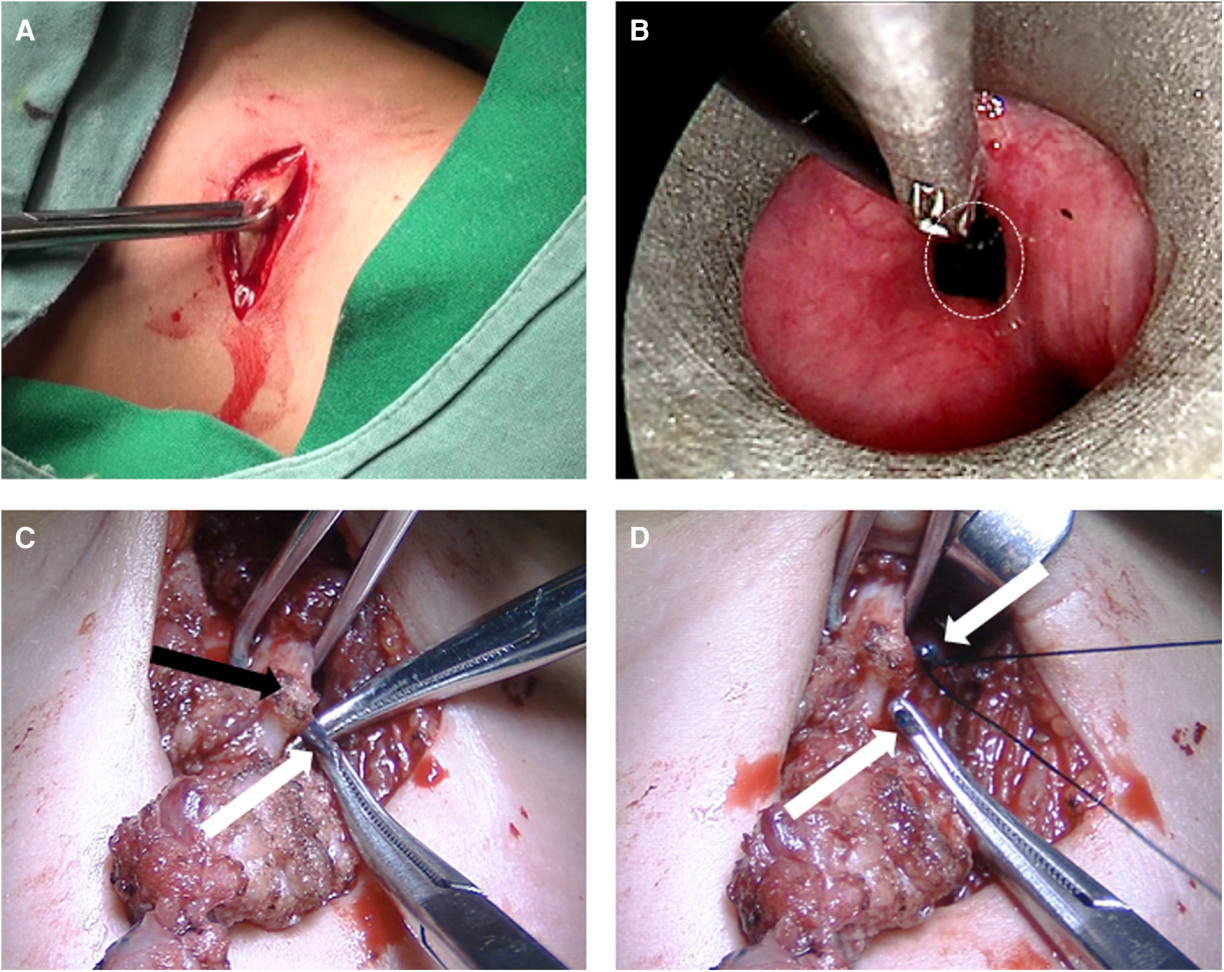
Intraoperative images of open surgical excision. (**A**) Neck fusiform incision; (**B**): methylene blue as an indicator; (**C**): the methylene-blue- dyed fistula under thyroid cartilage. (**D**) High ligation and resection of the fistulous tract. White circle: the internal opening; White arrow: fistula; Black arrow: laminae of thyroid cartilage.

OSE was performed by the chief surgeon (Dr. Li). The patients were placed supine under general anesthesia. The internal opening of CPSF was located under suspension laryngoscopy (GIF-160; Olympus, Tokyo, Japan). To locate the fistula tract, 1 ml methylene blue was injected into the internal opening as an indicator. Then, diluted iodine was used to disinfect surgical area and a 4–5 cm fusiform incision was made at the original scar or mass on the neck. After the division of subcutaneous tissue and the platysma muscles, the sternocleidomastoid and cervical strap muscles were isolated. The lesions were commonly tracked to the lateral to the superior pole of the thyroid and then the the inferior cornu of thyroid cartilage was separated to expose the methylene-blue-dyed fistula. When the fistula was fully dissected, high ligation and resection of the fistulous tract were conducted (Figure 2). Patients who underwent OSE typically have a nasogastric tube left in place for 3 days postoperatively.

### 2.3 Statistical analysis

All statistical analyses were performed using the SPSS 25.0 software program (Chicago, IL, USA). To assess the normality of the parameters, Kolmogorov–Smironoy analysis was conducted. For the analysis of differences between two groups, the independent sample t-test was applied for variables with a normal distribution, while the non-parametric Wilcoxon Mann-Whitney test was employed for variables with a skewed distribution. Categorical variables were evaluated using the *χ*^2^ test. A significance level of *P *< 0.05 was used to determine statistical significance.

## 3 Results

### 3.1 Clinical data

**Figure 3:**
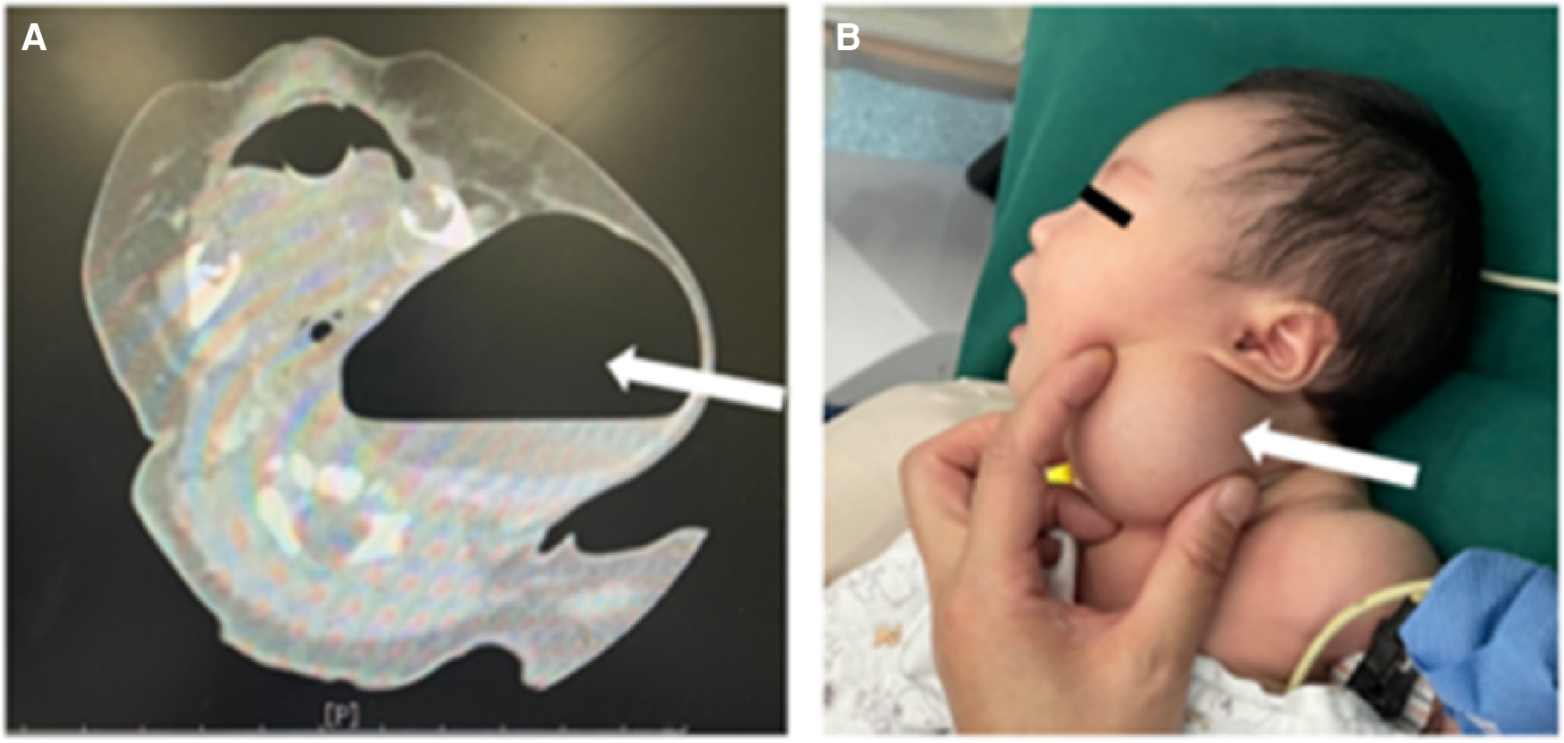
Giant piriform fossa cyst of newborn. (**A**) CT images; (**B**) clinical photograph. White arrow: giant piriform fossa cyst.

This study included 166 patients with CPSF (Table 1). Among them, there were 79 males and 87 females, the median age of whom was 4.8 years (8 days to 15 years). Among the total of 166 cases, 152 occurred on the left side (152/166, 91.6%), 12 occurred on the right side (12/166, 7.2%), and 2 occurred bilaterally (2/166, 1.2%). The main clinical manifestations of these patients included recurrent neck swelling (90 cases, 54.2%), neck abscess (65 cases, 39.2%), neck mass (3 cases, 1.8%), and suppurative thyroiditis or subacute thyroiditis (17 cases, 10.2%). Before surgery, 74 cases (74/166, 44.6%) were in AIS, while 92 cases (92/166, 55.4%) were in non-infection stage (NIS). Among them, 48 cases underwent OSE, while 118 cases underwent RA. In this study, 3 neonates with giant piriform fossa cyst underwent OSE (Figure 3). The average follow-up period was 27.7 months (ranging from 1.0 to 58.3 months). Due to the rarity of CPSF, in previous treatment, misdiagnosis occurred in 36 cases (36/166, 21.7%).

**Table 1:** Characteristics of patients with CPSF.

Characteristics	Number (%)
Gender	
Male	79 (47.6)
Female	87 (52.4)
Age at surgery, median (range)	4.8 years (8 days to 15 years)
Laterality	
Left	152 (91.6)
Right	12 (7.2)
Bilateral	2 (1.2)
Typical symptoms	
Repeated neck swelling	90 (54.2)
Neck abscess	65 (39.2)
Neck mass	3 (1.8)
Suppurative thyroiditis or subacute thyroiditis	17 (10.2)
Infection stage	
AIS	74 (44.6)
NIS	92 (55.4)
Imaging examinations	
Ultrasound	
Positive^a^	106 (73.6)
Negative	38 (26.4)
Plain scan and enhanced CT	
Positive^b^	106 (76.3)
Negative	33 (23.7)
Fiberoptic laryngoscopy	
Positive^c^	41 (56.9)
Negative	31 (43.1)
Surgical methods	
OSE	48 (28.9%)
RA	118 (71.1%)
Follow-up, median (range)	27.7 months (1.0–58.3 months)

AIS: acute infection stage, NIS: non-infection stage, CT: computed tomography, OSE: open surgical excision, RA: radiofrequency ablation.

^a^ Detectable fistula in the lateral lobe of thyroid (cable-like, tubular hypoechoic connected to body surface or subcutaneous), gas-echo in the upper area of the ipsilateral thyroid, or inflammatory-abscess formation, or mixed echo (uneven hypoechoic signal and low-echo area in the center).

^b^ Anomalous and uneven density soft tissue in the lower area of pyriform sinus, large cystic lesion with air and fluid and tubular structures inside the thyroid gland. On enhanced CT, the lesions tended to appear as heterogeneous and slight enhancement.

^c^ Confirmed internal opening of sinus tract.

### 3.2 Diagnostic methods

**Figure 4:**
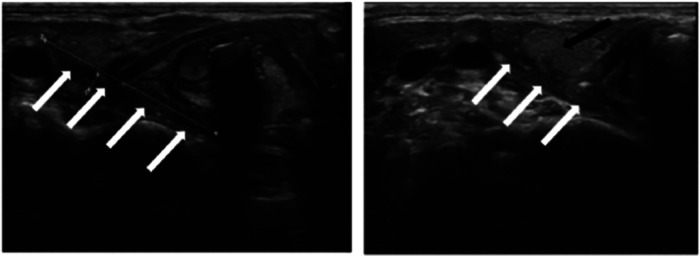
Ultrasound of neck. Tubular hypoechoic fistula into the posterior part of the right lobe of the thyroid gland. White arrow: fistula; Black arrow: thyroid gland.

**Figure 5:**
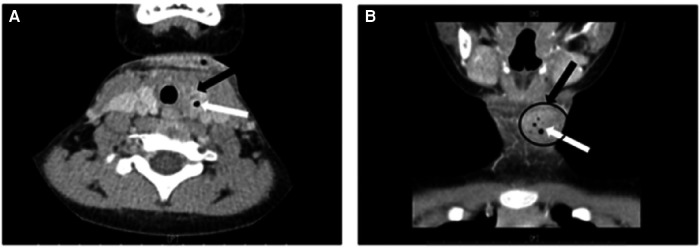
CT scan of neck. (**A**) Axial view; (**B**) coronal view. Black arrow and black circle: irregular low-density mass in the left lobe of the thyroid, consistent with a deep cervical abscess near the left thyroid; white arrow: point-like gas density shadow.

A total of 144 patients (86.7%) underwent neck ultrasound, while 139 patients (83.7%) underwent neck enhanced computed tomography (CT) scans. Additionally, fiberoptic laryngoscopy was performed on 72 patients (43.4%) prior to the operation (Table 1). The diagnostic rate of ultrasonography was 73.6% (106/144). The main manifestations in these cases included: detectable fistula in the lobe of thyroid (cable-like, tubular hypoechoic connected to body surface or subcutaneous), gas-echo in the upper area of the ipsilateral thyroid, or inflammatory-abscess formation, or mixed echo (uneven hypoechoic signal and low-echo area in the center) (Figure 4). 106 cases (76.3%) were diagnosed with CPSF by plain scan and enhanced CT, which revealed anomalous and uneven density soft tissue in the lower area of pyriform sinus, large cystic lesion with air and fluid and tubular structures inside the thyroid gland. On enhanced CT, the lesions tended to appear as heterogeneous and slight enhancement. Gas forming was another typical feature of CPSF (Figure 5). Fiberoptic laryngoscopy confirmed the presence of an internal opening of the sinus tract in 41 cases, and the detection rate was 56.9%.

### 3.3 Follow-up

In this research, OSE and RA were respectively performed in 48 cases and 118 cases (Table 2). In OSE group, previous treatments included oral or intravenous antibiotics in 47 cases (97.9%), I&D in 36 cases (76.6%) and operations in 7 cases (from outside institutes). The median length of hospital stay was 4 days (range, 2–11 days). Postoperative temporary hoarseness was reported in 4 cases, all of whom showed improvement within a few days after surgery. No complications like neck infection, neck mass and dysphagia happened in all cases. Only one case experienced a relapse after operation in this series, and was subsequently treated with RA, resulting in successful secondary treatment.

**Table 2:** Comparison of RA and OSE.

Features	OSE	RA	*P* value
Number	48	118	NA
Age at surgery	4.1 (2.5–7.0)	5.0 (3–7.4)	0.404
Median (quartile)			
Previous treatments			
Antibiotics, *n* (%)	47 (97.9%)	116 (98.3%)	0.865
I&D, *n* (%)			**<0.01**
0	12 (25.0%)	72 (61.0%)	** **
≥1 time	36 (75.0%)	46 (39.0%)	** **
Operation (RA or open resection), *n* (%)	7 (14.6%)	9 (7.6%)	0.169
Length of hospital stay (days) Median (quartile)	4 (3–5)	2 (2–3)	**<0.01**
Gastric tube indwelling (days)	3	14	NA
Postoperative complications, *n* (%)			0.230
Hoarseness	3 (6.3%)	16 (13.6%)	
Neck infection	0	1 (0.8%)	
Neck mass	0	0	
Dysphagia	0	0	
Recurrence, *n* (%)	1 (2.1%)	7(5.9%)	0.516

OSE, open surgical excision; RA, radiofrequency ablation; I&D, incision and drainage.

Bold values represent statistical differences.

In the RA group, previous treatments included oral or intravenous antibiotics in 116 cases (98.3%), and I&D in 46 cases (39.0%). The median length of hospital stay was 2 days (2–10 days). Postoperative temporary hoarseness occurred in 16 cases. There was one reported case of neck infection. 7 cases experienced a relapse after the operation and was then treated with conservative measures (1 cases), RA (1 cases) and OSE (5 cases); For 35 cases in AIS, I&D was simultaneously performed during operation.

## 4 Discussion

CPSF was initially reported by Sandborn in 1972, accounting for 3%–10% of all pharyngeal lesions (8). It is a rare congenital anomaly which originates from the incomplete embryonic development of the third or fourth branchial arch. Research has indicated that there is no prominent difference of the incidence rate between males and females (5). In this study, the male-to-female ratio was 1:1.1, which indicated no gender dominance. The predominance of CPSF occurring on the left side (over 90%) is likely due to the degeneration or absence of the right ultimobranchial body, or the asymmetrical development of blood vessels in the fourth branchial arch (3).

Children with CPSF often receive initial diagnoses of recurrent neck swelling neck abscess, neck mass, suppurative thyroiditis or subacute thyroiditis (3). Clinically, recurrent thyroiditis frequently occurs because the thyroid is likely to be invaded by bacteria through the fistula between the thyroid and internal opening of CPSF (9). In this series, the misdiagnosis rate in previous treatment before surgery reached 21.7%. For some patients who experienced repeated neck infections before receiving an accurate diagnosis, it is common for them to undergo excessive antibiotic treatment and multiple I&D or puncture drainage (5). Apart from the aforementioned inaccurate diagnoses, CPSF has been misidentified as mumps, esophageal fistula, lymphadenopathy, lymphangioma, second branchial fistula, tuberculosis (6), dermoid cyst, thyroglossal cyst, ectopic thyroid, diverticular rupture (10), neck cellulitis (11). In clinical practice, suppurative thyroiditis is a rare occurrence in children. In cases where suppurative thyroiditis is observed in children, it is crucial to maintain a high level of vigilance for infection caused by CPSF. Therefore, we strongly advise conducting the following examinations for children exhibiting recurrent neck swelling, neck abscess, neck mass, suppurative thyroiditis, or subacute thyroiditis, particularly on the left side, along with repeated drainage and delayed wound healing. These symptoms raise a high suspicion of CPSF in such cases.

The gold standard for diagnosing CPSF is the identification of internal opening of the sinus tract under suspension laryngoscope. However, this examination is not routinely performed due to the requirement for general anesthesia. Besides, fiberoptic laryngoscopy is recommended by some experts to locate the internal opening of the CPSF. Nevertheless, it is reported that the positive rate of fiberoptic laryngoscopy is relatively low, similarly to that in this study (56.9%), possibly due to the difficulty of complete anatomical exposure and unsatisfactory cooperation from children (3–5). Ultrasound is the preferred choice to repetitively detect the infammatory process and neck fistula in children, which is respectively shown as a hypoechoic mass or cord-like tubular structures (4, 5). However, ultrasound has limitations in assessing the depth and extent of CPSF. Instead, CT can be used to understand the anatomy and vital adjacent structures better (5). Researches have shown that enhanced CT estimates the length of the fistula and the positional relation between the fistula and adjacent structures like blood vessel and thyroid tissue (5). Based on our experience, we recommend that ultrasound, enhanced CT, fiberoptic laryngoscopy are the preferred diagnostic tools for CPSF. Depending on the available diagnostic equipment and the varying levels of expertise among radiologists and surgeons, these methods can be used individually or in combination to assist in the diagnosis.

For the treatment of CPSF in AIS, the conventional approaches are antibiotic treatment, I&D or puncture drainage. This study shows that 82 cases underwent I&D. One patient endured 7 infections and 5 I&D procedures prior to receive radical surgery. This highlights that conservative treatment following infection still poses a significant risk of reinfection.

The typical surgical method of CPSF is OSE in NIS, which should be performed 2–3 months after controlling the acute infection. Nevertheless, when patients suffer from frequently repeated infections, the surgery can be expedited to 1 month after the acute infection (4). Owing to the importance of the thyroid in the growth of children, we recommend that the thyroid tissue should be preserved during surgery even if the thyroid is invaded, as it does not pose a risk of recurrence (3). Theoretically, complete excision of the tract is considered as the most ideal and reliable treatment to prevent a recurrence. However, complete resection of the whole tract and high ligation of internal fistula are difficult and challenging. The small size and deep location of the fistula, as well as its proximity to the carotid sheath and important nerves, make the procedure difficult (3, 6). The normal anatomy here is prone to be damaged by recurrent infections and previous treatments, such as I&D and failed radical operations (4, 12). To accurately locate the fistula, several methods can be applied, including endoscopy-assisted methylene blue injection, intubation through the internal opening and light guided procedure (13, 14). In this data, the complication rate and the recurrence rate associated with OSE were both low (6.3%, 0.8%). Typically, the recurrence rate was lower than the rate of other reports (6, 15). The low recurrence rate and low incidence of vocal cord paralysis observed in this study can be attributed to the surgeon's precise understanding of the anatomical structures surrounding the fistula. By meticulously exploring the deep extent of the fistula, directly elevating the thyroid cartilage plate to expose the internal fistula marked with methylene blue through hypopharyngeal route, and performing accurate high-level ligation, the surgeon ensured the complete resection of the fistula.

With the development of endoscopic technique and popularization of minimally invasive surgery, endoscopic surgeries have been widely employed to treat CPSF, such as electrocauterization, chemocauterization, laser coagulation, fibrin glue and so on (7, 16–19). The objective of endoscopic cauterization is to induce scar tissue formation at the internal opening of CPSF, enabling adhesion and closure, thereby avoiding pharyngeal secretions, pathogen and food debris into the fistula. Nevertheless, research has revealed significant issues associated with these approaches, including high recurrent rate and severe complications (5, 12). In this study, we utilized endoscopic RA to avoid the inconvenience, inaccuracy and insecurity of other endoscopic techniques. Some experts believe that this technology can be considered as the primary treatment option for CPSF (12, 20).

Endoscopic RA technology utilizes a system of radiofrequency bipolar electrical current through a medium of normal saline, which generates a plasma field of sodium ions. Molecular bonds can be broken by these this plasma layer, leading to molecular dissociation and tissue ablation. One notable advantage of this technique is its ability to significantly reduce thermal damage to the sinus tract and adjacent structures by saline flushing and suction at lower temperatures (40℃–70℃), which is completely different from thermal injury caused by heat signatures (400℃–1,000℃) of electrocauterization and laser coagulation (5). As a minimally invasive and transoral surgery, this technology can avoid cervical scar and postoperative pain arising from external cervical incision. While some researchers suggest that endoscopic RA should not be performed during the acute or subacute stage of CPSF (4), our previous researches have shown that RA in combination with I&D of the abscess is a promising method for CPSF in AIS (5, 12). Abbas et al. (21) also reported a successful case of using RA in combination with I&D for a girl with a history of recurrent thyroid abscesses secondary to CPSF. Here is the analysis of transient hoarseness following RA (13.6%). During the ablation of the internal fistula opening using a plasma knife, the knife head is positioned on the medial aspect of the thyroid cartilage, causing thermal injury to the surrounding tissues, including the recurrent laryngeal nerve. Thus, it is critical to pay attention to the direction of the knife head during surgery to minimize damage to the recurrent laryngeal nerve. Therefore, based on our clinical experience during the 5 years, we consider the infectious period as an appropriate time for RA surgery, as it can effectively prevent the need for readmission and additional medication.

Some researchers have focused on the comparison between OSE and endoscopic cauterization. Hwang et al. (22) compared open surgery with endoscopic trichloroacetic acid chemocauterization, of which the recurrence rates were not significantly different (35.7% vs. 46.1%). Pu et al. (12) found that the success rate and postoperative temporary hoarseness rate in RA group were similar to those in surgical excision group (100% vs. 100%, 11.1% vs. 6.5%), while the risk of postoperative swelling and pain was higher in RA group (11.1% vs. 0%). There was no recurrence in both groups. In Chen's research (6), longer hospital stay was found in surgical excision group (10.50 ± 3.93 vs. 5.02 ± 3.30 days), while neck infection rate in this group was obviously lower than that in RA group (0% vs. 8.3%). Recurrence rates in these groups were not significantly different. In this data, we also found no significant disparities in postoperative complications and recurrence rates between OSE and RA (6.3% vs. 14.4%, 2.1% vs. 5.9% respectively). The duration of hospitalization was longer for patients who underwent OSE (Table 2). However, in comparison to OSE, the gastric tube retention time after RA was longer (3 days vs. 14 days). This extended duration aims to allow the internal fistula opening to fully heal after cauterization and prevent any food-related stimulation.

Therefore, in our experience, both OSE and RA are primary treatment options for CPSF, the selection of the surgical methods should be based on by individual conditions and clinical expertise of surgeons. Here is the summary of the indications for these two procedures:

1. RA is the preferred approach for pediatric CPSF, owing to its minimally invasive nature, shorter hospital stay, lower surgical complexity, and ease of operation. It is a safe method that avoids extensive damage. Moreover, RA can be performed even during the acute infection phase. For cases with abscess formation, simultaneous I&D can be performed, significantly reducing the treatment duration and eliminating the necessity for additional medication. Nonetheless, patients should be informed in advance about the potential complications following RA, such as temporary hoarseness and acute infection.
2. The following scenarios are recommended for open surgical intervention: (1) Newborns with massive piriform fossa cyst or masses in the neck; (2) Cases where previous RA procedures have been unsuccessful on two or more occasions; (3) Patients with evident scarring and inflammatory masses in the neck following previous unsuccessful I&D or open surgical procedures.

The limitations of this study are as follows. The presence of epithelial remnants and the persistence of a closed cavity following RA may give rise to long-term issues. Additionally, the retrospective nature of this study is another limitation. In consequence, it is essential to conduct a large-scale prospective clinical study with long-term follow-up.

## 5 Conclusion

CPSF is a rare congenital anomaly, over 90% of the fistula occurs on the left side, with occasional instances on the right side and bilaterally. Due to its rarity, CPSF should be considered in the differential diagnosis in children with recurrent neck infections, suppurative thyroiditis or subacute thyroiditis, accompanied by repeated drainage and long-delayed healing. Diagnostic tools such as ultrasound, enhanced CT and fiberoptic laryngoscopy are the preferred choices to aid preoperative diagnosis. Both OSE and RA are recognized as first-line treatment options for CPSF, which show no significant differences in prognosis, except hospitalization duration and the gastric tube retention time. Nevertheless, the indications for open surgical procedures and RA differ, which are influenced by factors such as the inflammatory stage, specific typing, previous treatments, and the surgeon's expertise. The selection of surgical approach should be carefully determined based on individual circumstances.

## Data Availability

The original contributions presented in the study are included in the article/Supplementary Material, further inquiries can be directed to the corresponding authors.
